# Quantitative assessment of mitochondrial DNA copies from whole genome sequencing

**DOI:** 10.1186/1471-2164-13-S7-S5

**Published:** 2012-12-07

**Authors:** Hsueh-Ting Chu, William WL Hsiao, Theresa TH Tsao, Ching-Mao Chang, Yen-Wenn Liu, Chen-Chieh Fan, Han Lin, Hen-Hong Chang, Tze-Jung Yeh, Jen-Chih Chen, Dun-Ming Huang, Chaur-Chin Chen, Cheng-Yan Kao

**Affiliations:** 1 Department of Biomedical informatics, Asia University, Taichung 41354, Taiwan; 2 Department of Computer Science and Information Engineering, Asia University, Taichung 41354, Taiwan; 3 BCCDC Public Health Microbiology & Reference Laboratory, Vancouver, BC, V5Z 4R4, Canada; 4 Department of Pathology and Laboratory Medicine, Vancouver, BC, V5Z 4R4, Canada; 5 Department of Computer Science and Information Engineering, National Taiwan University, Taipei 10617, Taiwan; 6 Graduate Institute of Clinical Medical Science, Chang Gung University, Taoyuan 33302, Taiwan; 7 Center for Traditional Chinese Medicine, Chang Gung Memorial Hospital at Taoyuan, Chang Gung Medical Foundation, Taoyuan 33302, Taiwan; 8 Institute of Food Science and Technology, National Taiwan University, Taipei 10617, Taiwan; 9 Institute of Biotechnology, National Taiwan University, Taipei 10617, Taiwan; 10 Department of Computer Science, National Tsing Hua University, Hsinchu 30013, Taiwan

## Abstract

**Background:**

Mitochondrial dysfunction is associated with various aging diseases. The copy number of mtDNA in human cells may therefore be a potential biomarker for diagnostics of aging. Here we propose a new computational method for the accurate assessment of mtDNA copies from whole genome sequencing data.

**Results:**

Two families of the human whole genome sequencing datasets from the HapMap and the 1000 Genomes projects were used for the accurate counting of mitochondrial DNA copy numbers. The results revealed the parental mitochondrial DNA copy numbers are significantly lower than that of their children in these samples. There are 8%~21% more copies of mtDNA in samples from the children than from their parents. The experiment demonstrated the possible correlations between the quantity of mitochondrial DNA and aging-related diseases.

**Conclusions:**

Since the next-generation sequencing technology strives to deliver affordable and non-biased sequencing results, accurate assessment of mtDNA copy numbers can be achieved effectively from the output of whole genome sequencing. We implemented the method as a software package MitoCounter with the source code and user's guide available to the public at http://sourceforge.net/projects/mitocounter/.

## Background

**Figure 1:**
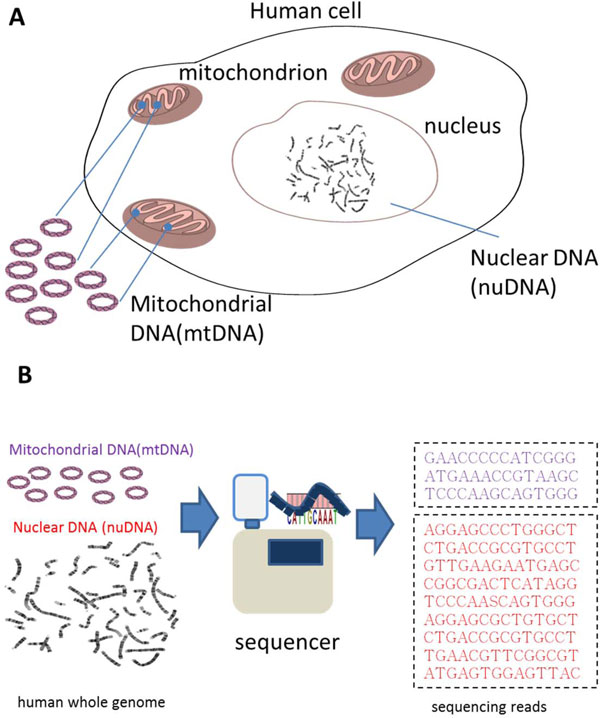
**Overview of human whole genome sequencing**. **A) **The human genome is composed of nuclear DNA and mitochondrial DNA. The nuclear DNA is stored on 23 chromosome pairs and there are multiple copies of small DNA located in mitochondria. **B) **The reads from the sequencing of human whole genome are mixed with both nuclear DNA and mitochondrial DNA.

Human mitochondria contain multiple copies of a 16.5 k bp, double-stranded, circular DNA molecule (Figure 1a). Since mitochondria are the organelles that generate chemical energy for cellular functions, many disease symptoms are linked to mitochondrial dysfunction, including poor growth, muscle weakness, hearing problems, visual problems, heart diseases, and liver diseases. There were many recent studies which showed significantly reduced mitochondrial DNA (mtDNA) copy numbers in cell samples of aging-related diseases [1-3]. A recent study also reported that mtDNA copy number is associated with cancer risk [4]. Therefore, quantitative assessment of mtDNA in human cells can elucidate the relationship between mitochondrial diseases and mitochondrial dysfunction.

In the past decade, quantitative real-time PCR assays were developed to estimate relative levels of mtDNA copy numbers in samples [2,5,6]. This approach measures the mtDNA copy number by determining the ratio of PCR amplicons to that of a single nuclear gene in experimental samples. The recent development of next-generation sequencing technology (NGS) revolutionized genomic studies and produced accurate whole genome sequencing (WGS) datasets [7]. As shown in Figure 1b, the output from human whole genome sequencing consists of both nuclear DNA (nuDNA) and mitochondrial DNA (mtDNA) molecules, thus it is convenient to assess mtDNA copy number from WGS dataset and can be an alternative to real-time PCR assays.

Here we demonstrate a computational method for counting mtDNA copy number using WGS datasets. The three steps in the process are (1) typing of mtDNA, (2) separation of mtDNA reads, and (3) calculation of mtDNA count. We developed a freely available software package called MitoCounter for this purpose. MitoCounter can be used to calculate the average copy numbers of mtDNA molecules in the sequenced samples. Besides, the separated mtDNA reads provide further analysis of mtDNA heteroplasmy. The mtDNA heteroplasmy represents the mixture of individual mtDNA mutations. Heteroplasmy levels can alter the clinical penetrance of primary mtDNA diseases [8,9].

## Methods

### A computational assay for counting mtDNA copies from a WGS dataset

Since the library construction bias is minimized with the next-generation sequencing platform [10], both mitochondrial DNA (mtDNA) and nuclear DNA (nuDNA) are sequenced together with equal opportunities. The output dataset comprises a mixture of mtDNA reads and nuDNA reads. Let the total number of nucleotide bases in the nuclear genome be 2N (for diploid chromosomes) and the number of bases in a mitochondrial DNA is M. Then the summation of nucleotide bases in the entire human genome is 2N+kM, where k is the number of mtDNA copies. The numbers of reads from nuDNA and the number of reads from mtDNA should reflect the ratio of 2N:kM.

That is,

(1) $$\frac{mtBases}{allBases}=\frac{kM}{2N+kM}$$

where *mtBases *is the total bases of sequenced reads from mtDNA and *allBases *is total bases of all sequenced reads from the output of a WGS procedure.

From an entire dataset of human whole genome sequencing, we separate the mtDNA reads from the others. Then the number of mtDNA copies can be approximated as

(2) $$k=\frac{mtBases*2N}{\left(allBases-mtBases\right)*M}$$

The equation for counting mtDNA copies is not suitable for plants (e.g. Arabidopsis) since their mtDNA sequences may contain segments of nuclear DNA. Besides, there are usually other DNA molecules in their cells, such as chloroplast genome and plasmid genome.

### Software implementation

In order to precisely separate mtDNA reads from a WGS dataset, it is necessary to determine the genotype of the mitochondrial genome first. We designed a program WgsMitoAssembler to identify the homoplasmic sequences, which present the inherent mutations in most of mtDNA molecules. The program WgsMitoAssembler is a guided assembler, and it requires a reference mitochondrial sequence which is used to choose a beginning read and an ending read from the entire WGS dataset. We use the reference mtDNA sequence (GenBank: NC_001807.4) for the purpose. We then search for best candidate reads which can extend the beginning read from the 3' end to the 5' end until the ending read is met.

**Figure 2:**
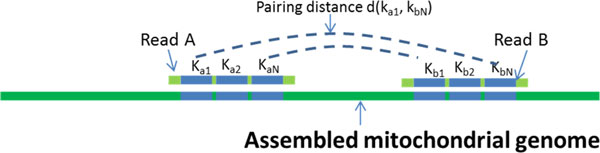
**An error-tolerant mapping algorithm for filtering mitochondrial reads**.

After the typing of the target mitochondrial genome, the homoplasmy sequence is used in the second program WgsMitoCounter. The program performs the job of separating mitochondrial reads from the entire WGS dataset. Considering that some of sequenced reads may contain erroneous bases, we design an error-tolerant mapping algorithm shown in Figure 2. We search for sub-sequences of paired reads which are indexed as mtDNA fragments and the accuracy of mapping is determined by the pairing distances. WgsMitoCounter will output a CSV file which records the number of mitochondrial reads in each run of the entire dataset. The template of final calculation for mtDNA copy number is provided in Additional file 1.

## Results and discussion

### Parental mtDNA samples have less copy numbers

We apply the analysis to public WGS datasets from the HapMap [11] and 1000 Genomes [12] projects. We chose six high-coverage WGS datasets for two pedigree trios: YOR009 and CEPH146 and two low-coverage WGS datasets for individual elders (Sample ID: NA11831 and NA06985), listed in Table 1. YOR009 is an African family. CEPH1463 is a family from Utah with Northern and Western European ancestry. The two individuals are also from the CEPH population and were recorded as the grandparents in the 1000 Genomes project. These DNA samples were isolated from B-lymphocyte cells derived from blood. Table 2 lists the results of counting mitochondrial DNA on the selected datasets. The mtDNA counts for the YOR009 family are between 645~752 and for CEPH1463 family are between 734~950. Besides, the mtDNA counts for the two individual elders are 662 and 755.

**Table 1:** Typing of mtDNA from whole genome sequencing samples

HapMap Family	Sample	Sex	Relation	mtDNA Length	Haplogroup* Reference	Haplogroup
YOR009	NA18507	male	father	16567 bp	AF346986	L1b
	NA18508	female	mother	16567 bp	DQ341073	L3b
	NA18506	male	child	16567 bp	DQ341073	L3b
CEPH146	NA12891	male	father	16572 bp	EU715237	H1
	NA12892	female	mother	16570 bp	GU945543	H13a1a1
	NA12878	female	child	16570 bp	GU945543	H13a1a1

CEPH1350	NA11831	male	grandfather	16569 bp	AY495174	H5
CEPH1341	NA06985	female	grandmother	16569 bp	AY882388	U4b

* The references for haplogroup are selected from top BLAST results.

**Table 2:** Counts of mtDNA from whole genome sequencing samples

Sample	Relation	SRA ID of dataset	Runs in dataset	Total bases	mtDNA bases	mtDNA Ratio	mtDNA count
NA18507	father	ERX009609	24	135.2G	249.8M	0.185%	646.84
NA18508	mother	ERX009610	24	133.2G	239.7M	0.180%	629.69
NA18506	child	ERX009608	24	132.3G	273.0M	0.206%	722.10
NA12891	father	ERX000172	35	1.538G	36.78M	0.239%	837.11
NA12892	mother	ERX000174	42	1.543G	31.58M	0.205%	716.25
NA12878	child	ERX000170	55	2.762G	71.92M	0.260%	911.38

NA11831	grandfather	SRX116265	1	4.15G	7.54M	0.182%	662.74
NA06985	grandmother	SRX116266	1	11.97G	24.80M	0.207%	755.92

For the counting results of these WGS samples (Additional file 2 and 3), ANOVA analysis revealed significant differences among the mtDNA counts within each family group: for YOR009, *F*(2,69) = 916.01, *p *= 2.06E-50 and for CEPH1463, *F*(2,169) = 58.75, *p *= 7.26632E-19. It showed that the offspring had 8%~23% more mtDNA than their parents in these samples. Although we did not investigate the possible artefacts caused by sequencing procedures, the results consistently demonstrated that there are more mtDNA sequences within younger persons' lymphocyte cells.

## Conclusions

Many studies suggested that mitochondrial functions become defective as we age. Recent findings suggests that structural changes in mitochondria, including increased mitochondrial fragmentation and decreased mitochondrial fusion, are critical factors associated with mitochondrial dysfunction and cell death in aging and neurodegenerative diseases [13,14]. Therefore, the proposed quantitative analysis of mtDNA can help to further elucidate the dynamics of mitochondrial diseases. It is expected that cost for sequencing personal whole genome will be less than $1000 in the near future. For the purpose of counting mitochondrial DNA, it only requires a low coverage of the whole genome and the cost may be further reduced to $50. The cost-effectiveness of the procedure makes the proposed method of counting mitochondrial DNA as a useful diagnostic tool to study aging and aging-related diseases for individuals.

## Availability and requirements

In the MitoCounter software package, both the programs WgsMitoAssembler and WgsMitoCounter were implemented in C# with the .NET Framework which can be run on 64-bit Windows. The program WgsMitoCounter requires paired-end WGS datasets from Illumina sequencing platform. The MitoCounter software with a user manual is available at the Web site: http://sourceforge.net/projects/mitocounter/

## Competing interests

The authors declare that they have no competing interests.

## Authors' contributions

HTC devised the method and wrote the software. HTC, WLH, TTT, CMC, YWL, CCF, HHC, HL, TJY, JCC, DMH and CCC discussed the project and jointly wrote the manuscript. CYK conceived the project.

## Supplementary Material

Additional File 1 **Supplementary Software**. The MitoCounter software package consists of two execution programs, an Excel template and a User manual. Click here for file

Additional File 2 **Supplementary Table 1**. An Excel file lists the ratio of mitochondrial reads in each WGS run for the family trio YOR009 samples. Click here for file

Additional File 3 **Supplementary Table 2**. An Excel file lists the ratio of mitochondrial reads in each WGS run for the family trio CEPH1463 samples. Click here for file
