# Supplementary material for: Quantitative assessment of mitochondrial DNA copies from whole genome sequencing
Source: BMC Genomics. 2012 Dec 7;13(Suppl 7):S5. doi: 10.1186/1471-2164-13-S7-S5 (PMC3521385; doi:10.1186/1471-2164-13-S7-S5)

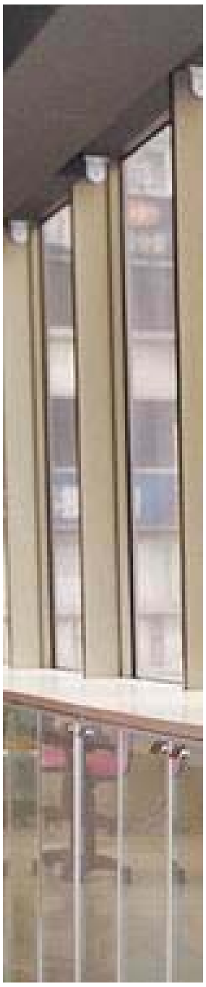

MitoCounter

User's Guide

Counting mitochondrial DNA in cells

Hsueh-Ting Chu
Htchu.taiwan@gmail.com

Feb., 2012

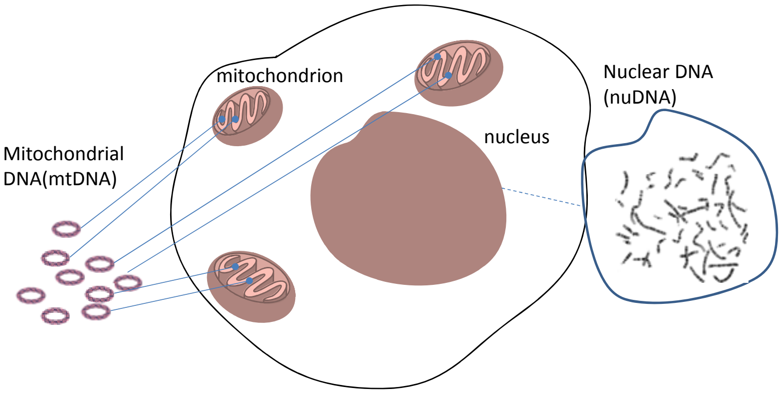

USER'S MANUAL

TABLE OF CONTENTS

Page #

Step 1: Prepare Whole Genome Sequencing Dataset ... *1*

1.1 Check the dataset ERX009608 at the European Nucleotide Archive ... 1

1.2 Download all the runs in the dataset through FTP ... 2

1.3 Download the MitoCounter package from Sourceforge.net ... 2

Step2. Typing by WGSMitoAssembler ... *3*

2.1 Mapping assembly: Reference + Program + Sequenced Reads ... 3

2.2 Do assembly ... 4

2.3 Finish of assembly ... 4

2.4 Check the result ... 5

2.5 Check haplogroup by BLAST ... 6

Step3: Counting by WGSMitoCounter ... *7*

3.1 Genome + Program + Sequenced Reads ... 7

3.2 Do Counting ... 8

3.3 Check the result ... 9

3.4 Calculation copy number ... 10

Revision Sheet

Release No.	Date	Revision Description
Rev. 0	2/10/02	User's Guide

STEP 1: PREPARE WHOLE GENOME SEQUENCING DATASET

1.1 Check the dataset ERX009608 at the European Nucleotide Archive

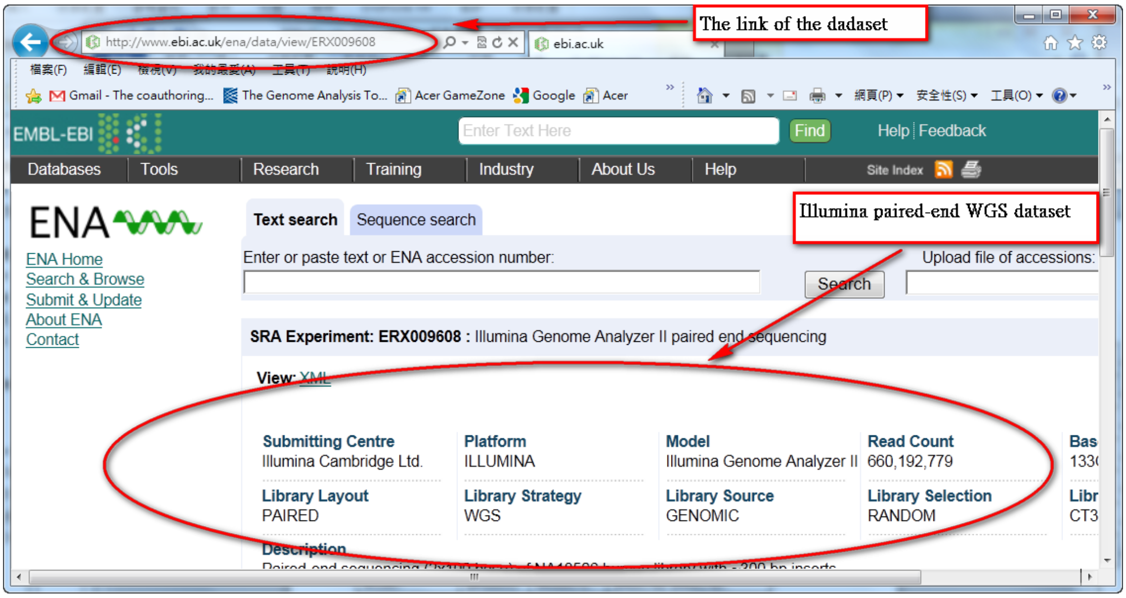

Study	Sample	Run	Organism	Instrument Model	Library Layout	Run Read Count	Run Base Count	ftp	Aspera	Galaxy
ERP000459	SRS003565	ERR024139	Homo sapiens	Illumina Genome Analyzer II	PAIRED	26,826,505	5Gb	Fastq file#1	not installed	Fastq file#1
ERP000459	SRS003565	ERR024139	Homo sapiens	Illumina Genome Analyzer II	PAIRED	26,826,505	5Gb	Fastq file#2	not installed	Fastq file#2
ERP000459	SRS003565	ERR024140	Homo sapiens	Illumina Genome Analyzer II	PAIRED	27,157,395	5Gb	Fastq file#1	not installed	Fastq file#1
ERP000459	SRS003565	ERR024140	Homo sapiens	Illumina Genome Analyzer II	PAIRED	27,157,395	5Gb	Fastq file#2	not installed	Fastq file#2
ERP000459	SRS003565	ERR024141	Homo sapiens	Illumina Genome Analyzer II	PAIRED	27,371,447	5Gb	Fastq file#1	not installed	Fastq file#1
ERP000459	SRS003565	ERR024141	Homo sapiens	Illumina Genome Analyzer II	PAIRED	27,371,447	5Gb	Fastq file#2	not installed	Fastq file#2
ERP000459	SRS003565	ERR024142	Homo sapiens	Illumina Genome Analyzer II	PAIRED	27,624,257	5Gb	Fastq file#1	not installed	Fastq file#1
ERP000459	SRS003565	ERR024142	Homo sapiens	Illumina Genome Analyzer II	PAIRED	27,624,257	5Gb	Fastq file#2	not installed	Fastq file#2
ERP000459	SRS003565	ERR024143	Homo sapiens	Illumina Genome Analyzer II	PAIRED	27,010,899	5Gb	Fastq file#1	not installed	Fastq file#1
ERP000459	SRS003565	ERR024143	Homo sapiens	Illumina Genome Analyzer II	PAIRED	27,010,899	5Gb	Fastq file#2	not installed	Fastq file#2

Next

1.2 Download all the runs in the dataset through FTP

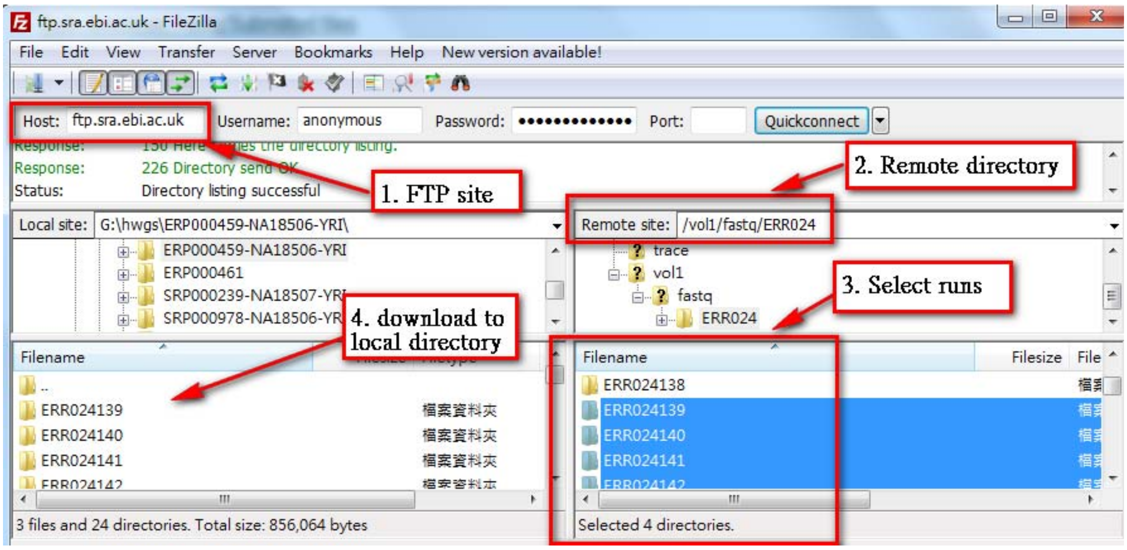

Table 1. List of runs in each Illumina WGS datasets

Samples	Study ERP	ERX	Runs
**NA18507**	ERP000460	ERX009609	ERR024163- ERR024186
**NA18508**	ERP000459	ERX009608	ERR024139- ERR024162
**NA18506**	ERP000461	ERX009610	ERR024201- ERR024200
**NA12891**	SRP000032	ERX000172	ERR001785- ERR001793
**NA12892**	SRP000032	ERX000174	ERR001827-ERR001868
**NA12878**	SRP000032	ERX000170	ERR001698-ERR002356

1.3 Download the MitoCounter package from Sourceforge.net

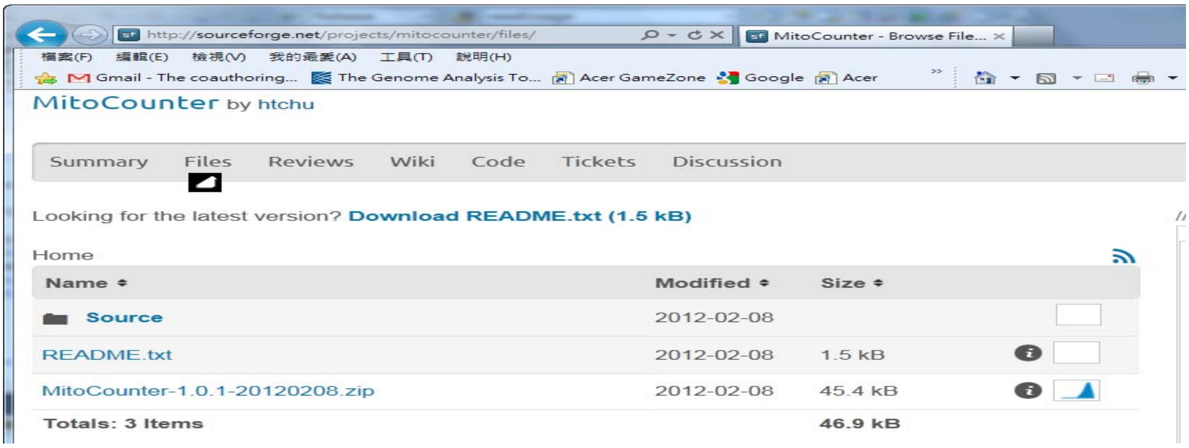

STEP2. TYPING BY WGSMITOASSEMBLER

2.1 Mapping assembly: Reference + Program + Sequenced Reads

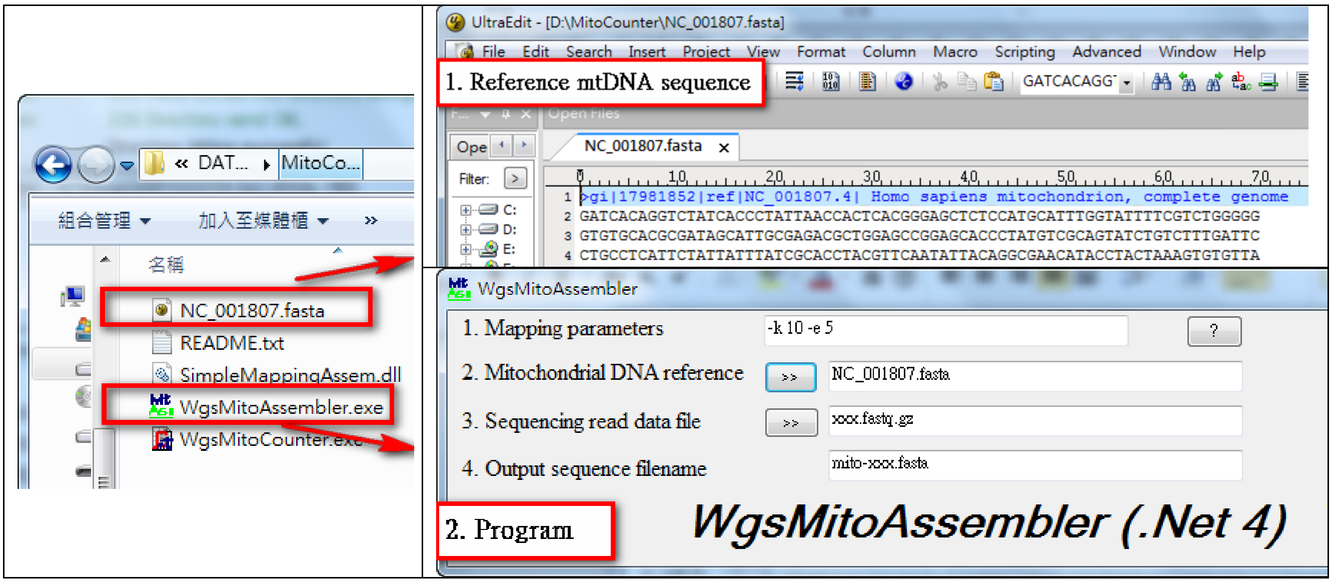

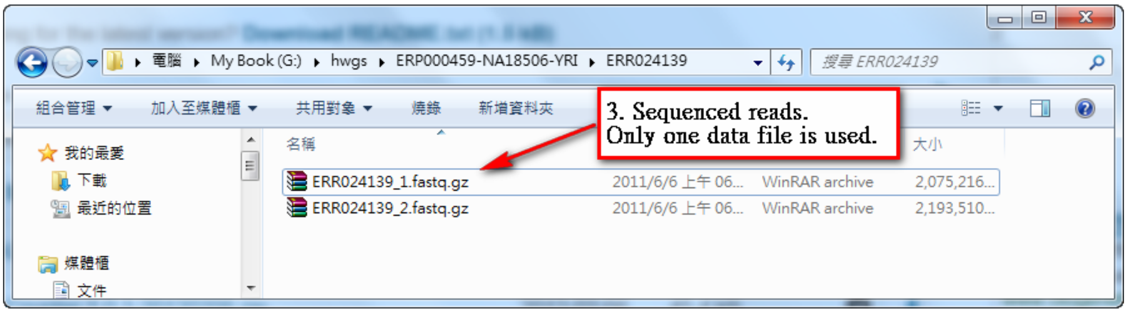

2.2 Do assembly

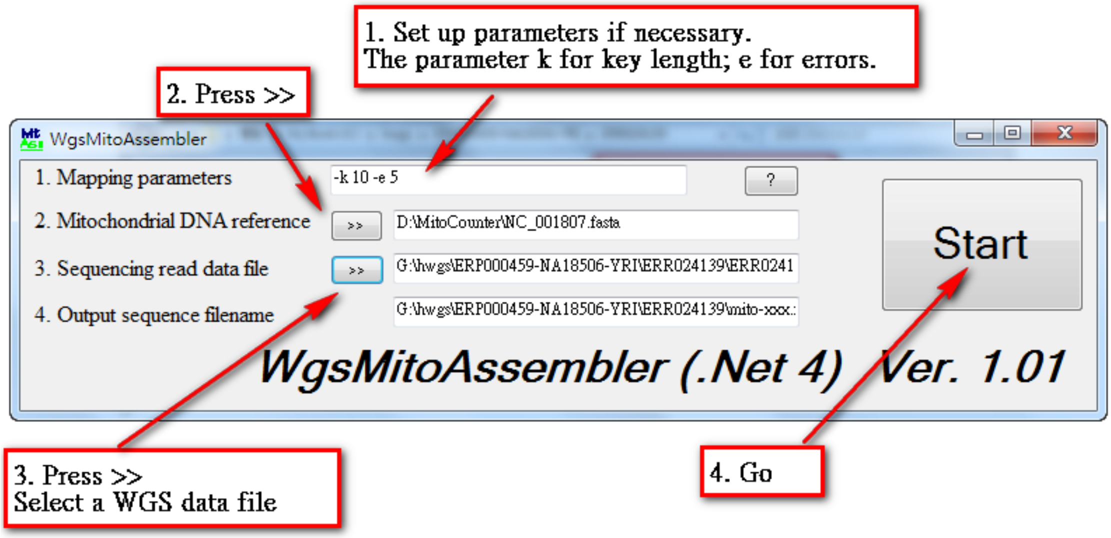

2.3 Finish of assembly

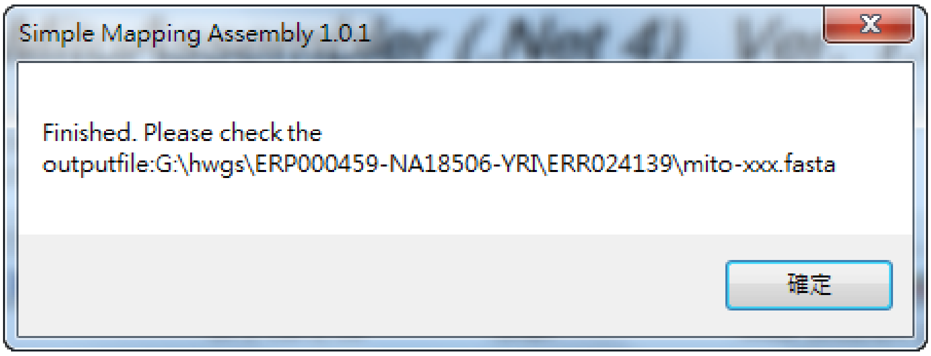

2.4 Check the result

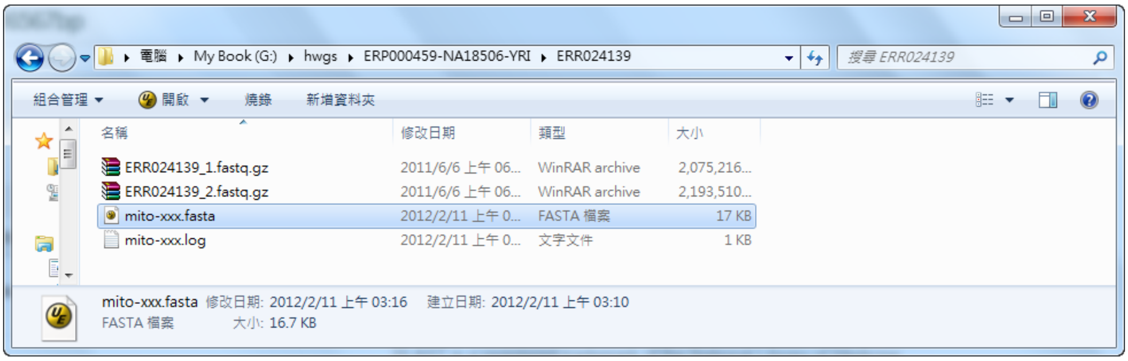

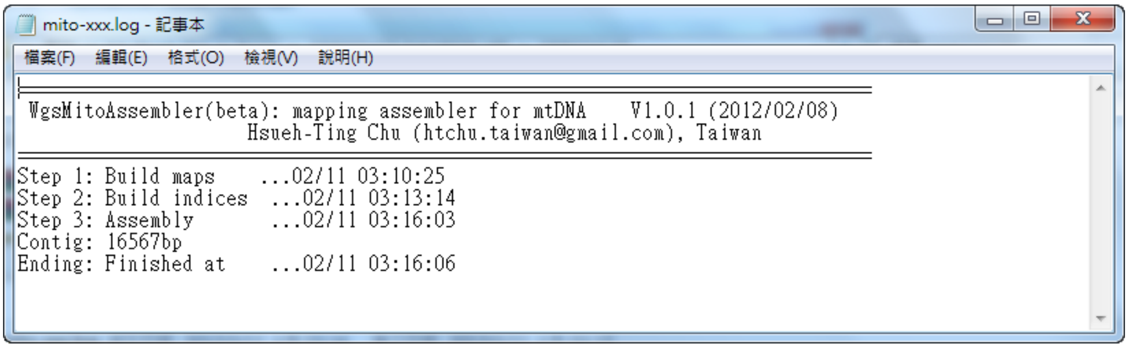

2.5 Check haplogroup by BLAST

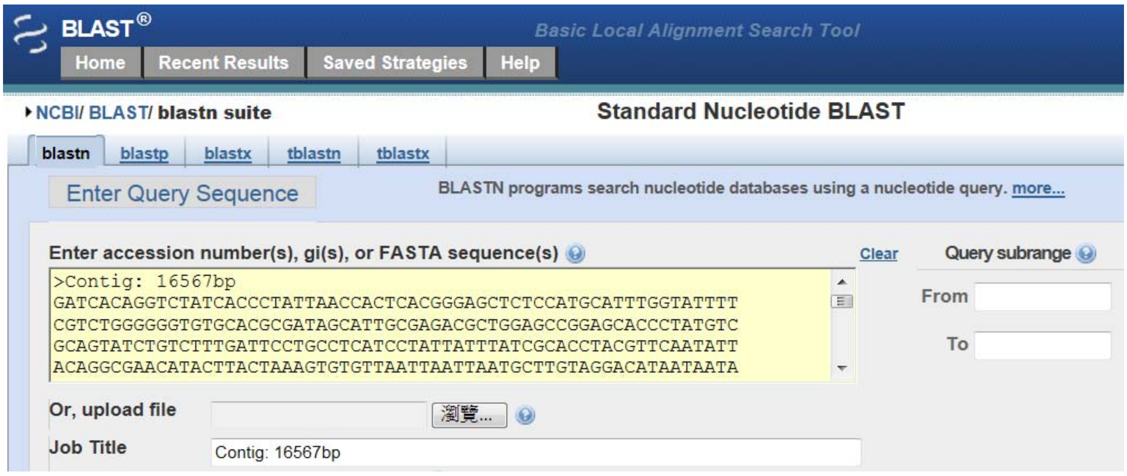

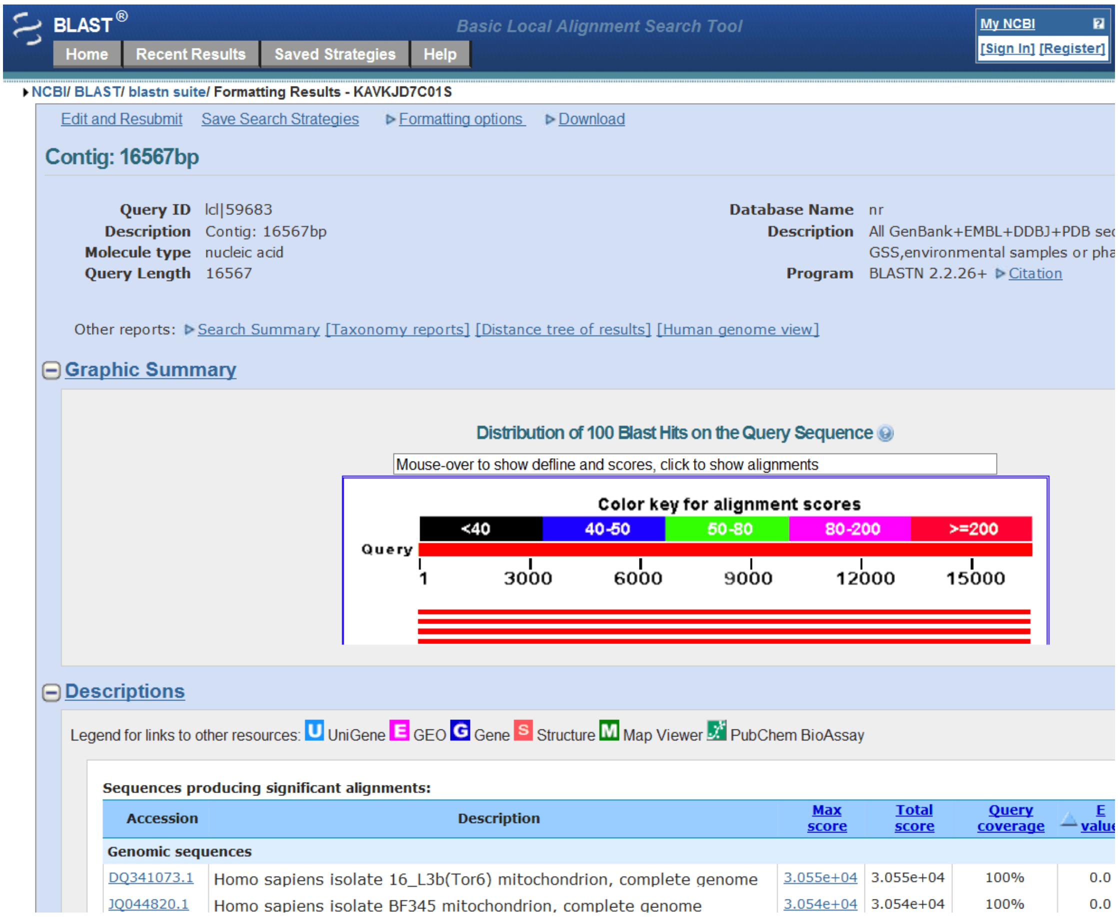

STEP3: COUNTING BY WGSMITOCOUNTER

3.1 Genome + Program + Sequenced Reads

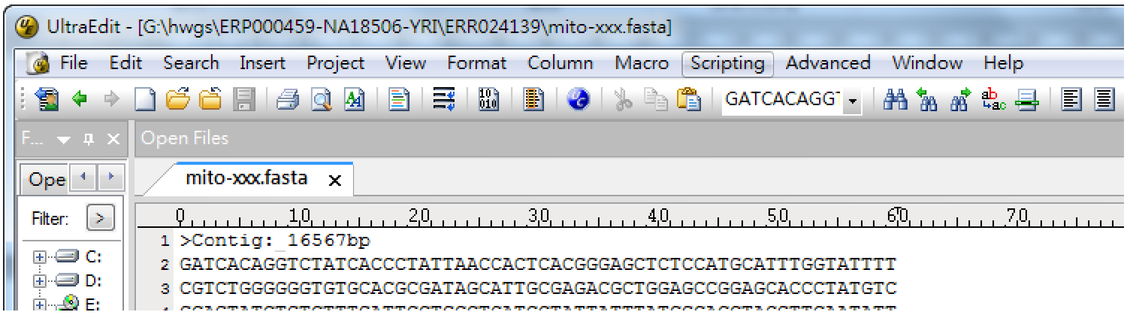

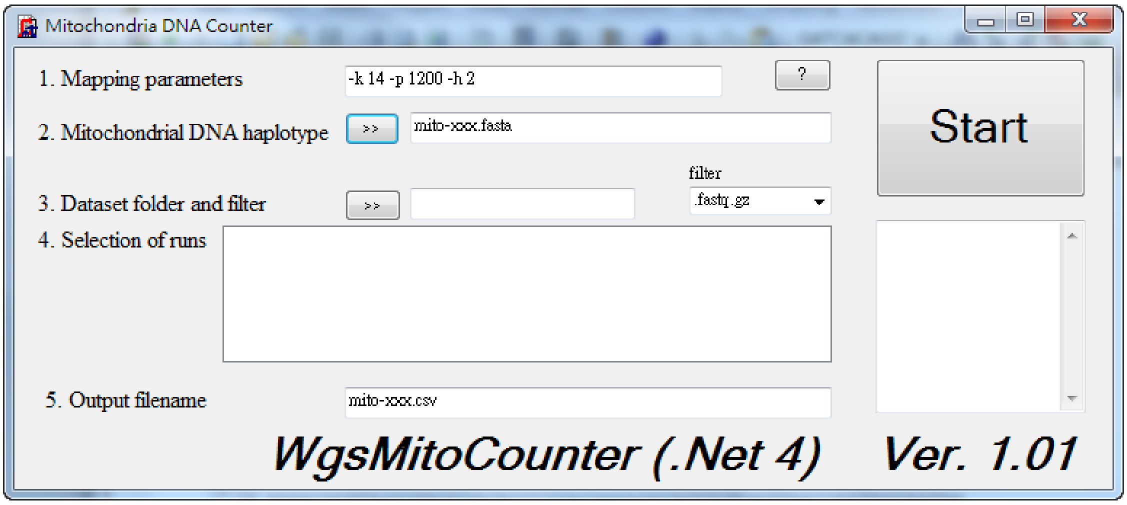

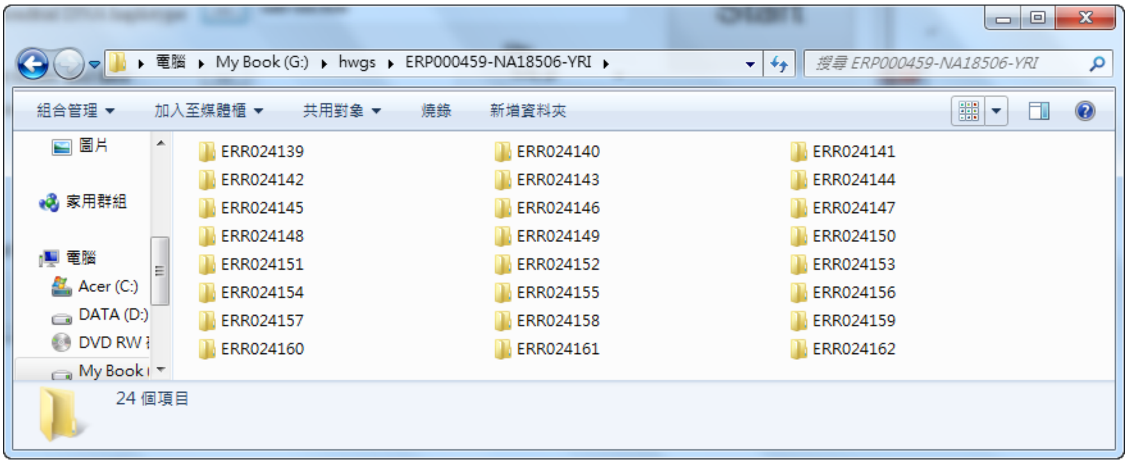

3.2 Do Counting

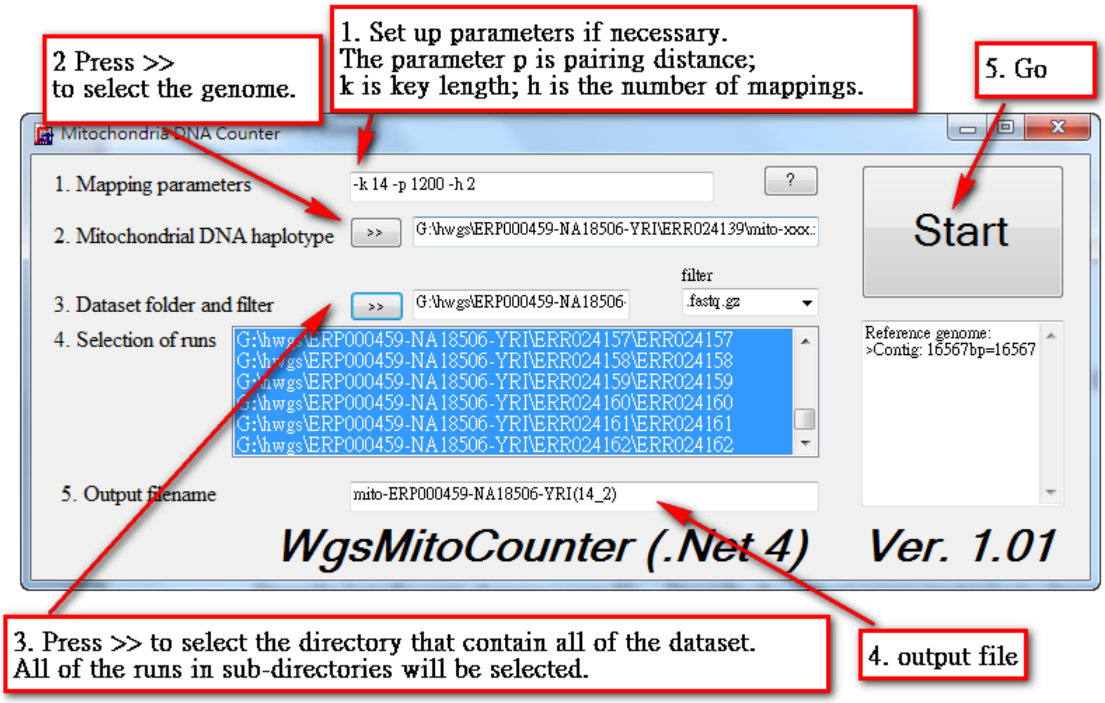

Table 2. Suggestion of parameters

Read length	Key length (-k)	Number of maps (-h)	Pairing distance
**30**	11	2	Depend on insert size
**36**	12	2	
**50**	12	3	
**100**	14	3	

3.3 Check the result

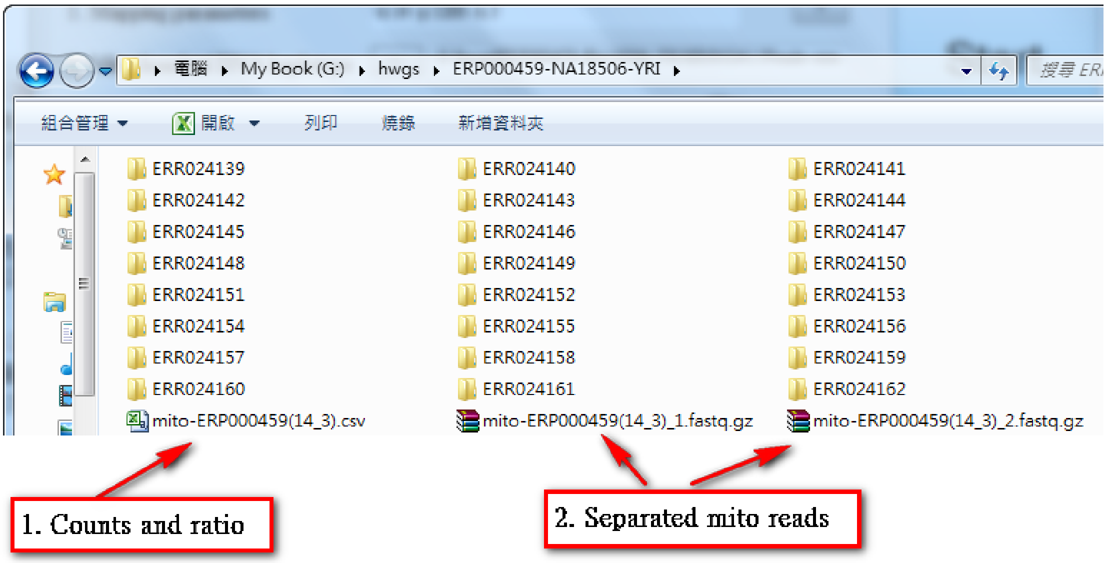

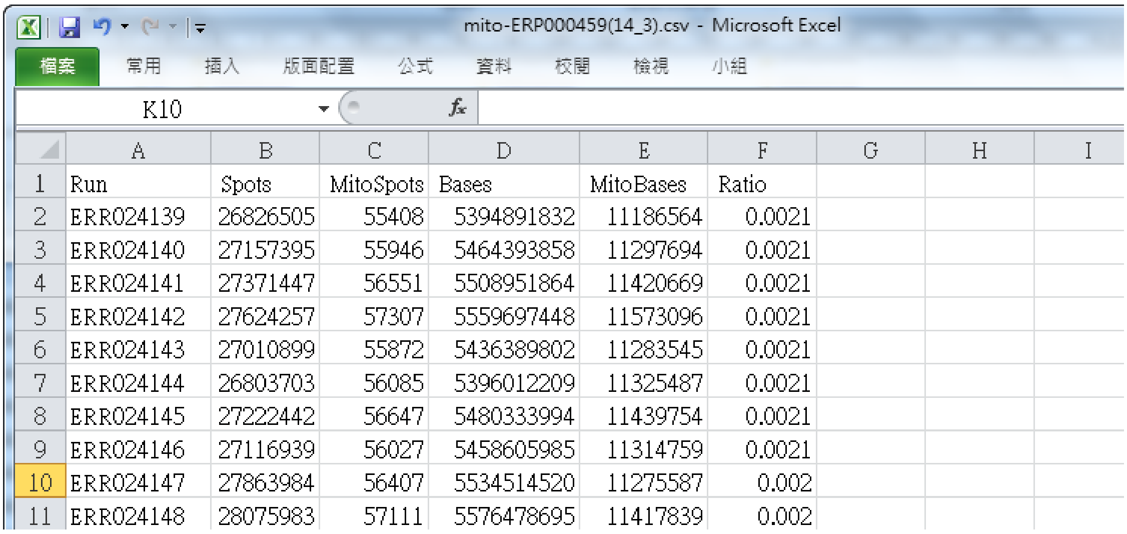

	A	B	C	D	E	F	G	H	I
1	Run	Spots	MitoSpots	Bases	MitoBases	Ratio			
2	ERR024139	26826505	55408	5394891832	11186564	0.0021			
3	ERR024140	27157395	55946	5464393858	11297694	0.0021			
4	ERR024141	27371447	56551	5508951864	11420669	0.0021			
5	ERR024142	27624257	57307	5559697448	11573096	0.0021			
6	ERR024143	27010899	55872	5436389802	11283545	0.0021			
7	ERR024144	26803703	56085	5396012209	11325487	0.0021			
8	ERR024145	27222442	56647	5480333994	11439754	0.0021			
9	ERR024146	27116939	56027	5458605985	11314759	0.0021			
10	ERR024147	27863984	56407	5534514520	11275587	0.002			
11	ERR024148	28075983	57111	5576478695	11417839	0.002			

3.4 Calculation of copy number

Check the last data row in the output file.

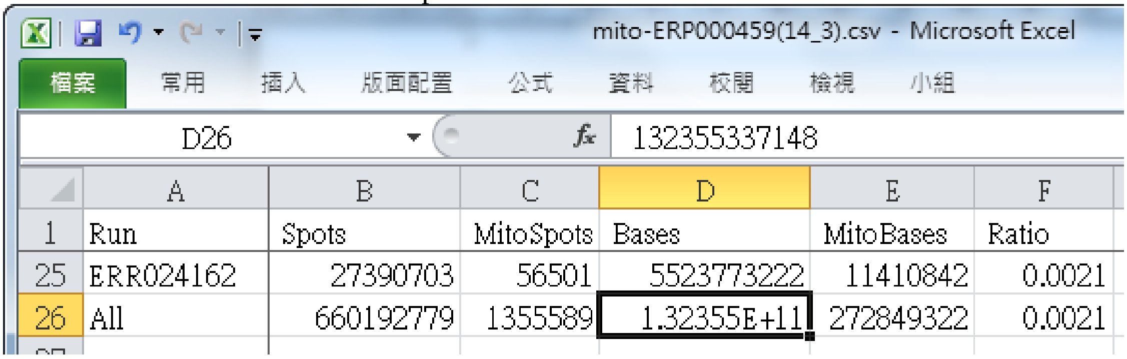

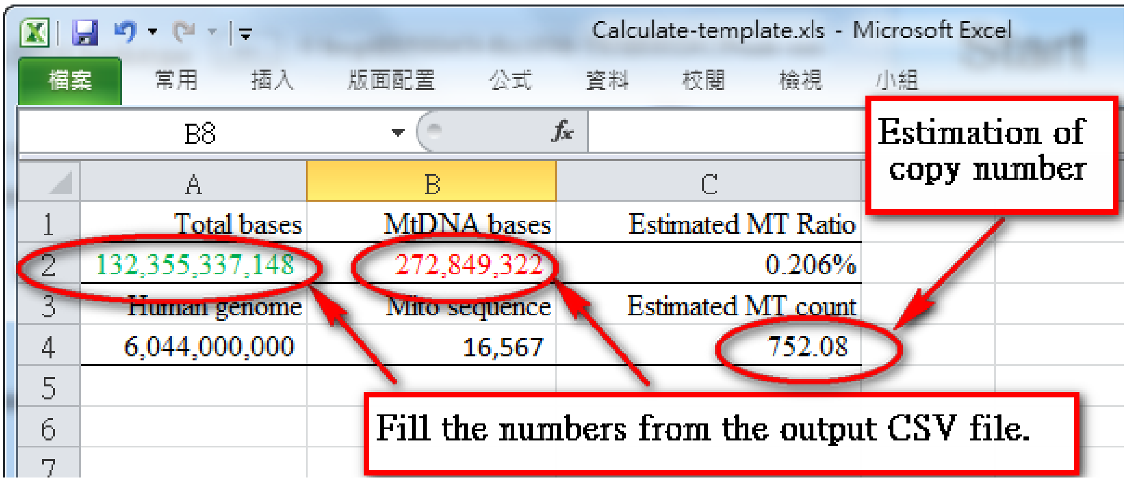

Supplement: Additional File 3 — Supplementary Table 2. An Excel file lists the ratio of mitochondrial reads in each WGS run for the family trio CEPH1463 samples. [file 1471-2164-13-S7-S5-S3.zip › MitoCounter-1.0.1-20120720/MitoCounterManual.pdf]
